# Adaptation to tolerate high doses of arabinoxylan is associated with fecal levels of *Bifidobacterium longum*

**DOI:** 10.1080/19490976.2024.2363021

**Published:** 2024-06-11

**Authors:** Edward C. Deehan, Zhengxiao Zhang, Nguyen K. Nguyen, Maria Elisa Perez-Muñoz, Janis Cole, Alessandra Riva, David Berry, Carla M. Prado, Jens Walter

**Affiliations:** a Department of Agricultural, Food and Nutritional Science, University of Alberta, Edmonton, Canada; b Department of Food Science and Technology, University of Nebraska, Lincoln, NE, USA; c Nebraska Food for Health Center, University of Nebraska, Lincoln, NE, USA; d Department of Medicine, University of Alberta, Edmonton, Canada; e College of Food and Biological Engineering, Jimei University, Xiamen, China; f Metabolism and Nutrition Research Group (MNUT), Louvain Drug Research Institute (LDRI), UCLouvain, Université catholique de Louvain, Brussels, Belgium; g Walloon Excellence in Life Sciences and Biotechnology (WELBIO), WEL Research Institute, Wavre, Belgium; h Centre for Microbiology and Environmental Systems Science, Department of Microbiology and Ecosystem Science, Division of Microbial Ecology, University of Vienna, Vienna, Austria; i Chair of Nutrition and Immunology, School of Life Sciences, Technical University of Munich, Freising-Weihenstephan, Germany; j Joint Microbiome Facility of the Medical University of Vienna, University of Vienna, Vienna, Austria; k Department of Biological Sciences, University of Alberta, Edmonton, Canada; l APC Microbiome Ireland, School of Microbiology, and Department of Medicine, University College Cork – National University of Ireland, Cork, Ireland

**Keywords:** Adults, arabinoxylan, *Bifidobacterium*, dietary fiber, gastrointestinal symptoms, gut microbiome, short-chain fatty acids, tolerance

## Abstract

Dietary fiber supplements are a strategy to close the ‘fiber gap’ and induce targeted modulations of the gut microbiota. However, higher doses of fiber supplements cause gastrointestinal (GI) symptoms that differ among individuals. What determines these inter-individual differences is insufficiently understood. Here we analyzed findings from a six-week randomized controlled trial that evaluated GI symptoms to corn bran arabinoxylan (AX; *n* = 15) relative to non-fermentable microcrystalline cellulose (MCC; *n* = 16) at efficacious supplement doses of 25 g/day (females) or 35 g/day (males) in adults with excess weight. Self-reported flatulence, bloating, and stomach aches were evaluated weekly. Bacterial taxa involved in AX fermentation were identified by bioorthogonal non-canonical amino acid tagging. Associations between GI symptoms, fecal microbiota features, and diet history were systematically investigated. AX supplementation increased symptoms during the first three weeks relative to MCC (*p* < 0.05, Mann-Whitney tests), but subjects ‘adapted’ with symptoms reverting to baseline levels toward the end of treatment. Symptom adaptations were individualized and correlated with the relative abundance of *Bifidobacterium longum* at baseline (r_s_ = 0.74, *p* = 0.002), within the bacterial community that utilized AX (r_s_ = 0.69, *p* = 0.006), and AX-induced shifts in acetate (r_s_ = 0.54, *p* = 0.039). Lower baseline consumption of animal-based foods and higher whole grains associated with less severity and better adaptation. These findings suggest that humans do ‘adapt’ to tolerate efficacious fiber doses, and this process is linked to their microbiome and dietary factors known to interact with gut microbes, providing a basis for the development of strategies for improved tolerance of dietary fibers.

## Introduction

Dietary fiber is an important dietary component for the prevention of chronic diseases^1,2^ and to ensure gut microbiome diversity and metabolic functionality.^3^ Governmental and nutritional organizations encourage consumption of fiber-rich whole foods to achieve recommended intakes of 25–38 g/day.^4–6^ However, fiber intake in socioeconomically developed societies has remained at only half of what is recommended,^5,7^ resulting in a ‘fiber gap’.^4^ We and others have argued that the fiber gap might be even larger in light of the amounts of dietary fiber consumed throughout most of human evolution, which is likely to have led to adaptations in human physiology and host-microbiome symbiosis to elevated levels of fiber.^8–10^ Thus, suggestions have been made that higher doses of dietary fiber may be necessary for consistent health benefits,^3,11^ a notion supported by systematic reviews and meta-analyses.^1,12,13^ Dietary fibers also offer exciting prospects for selective, targeted, and personalized modulations of gut microbiota composition and metabolic functions relevant to health,^14–17^, but physiologically relevant changes to the gut microbiome require higher doses.^18,19^ Purified fibers can be used in foods or as supplements to close the fiber gap,^8,10,20^ but it remains unknown whether modern humans would tolerate ancestral amounts of fiber. Even amounts required for consistent health benefits and/or to induce physiologically relevant changes to the gut microbiota induce symptoms in a subset of humans.^18^

Dietary fibers remain largely intact until they reach the colon where, dependent on chemical structure, some of them undergo fermentation by the microbiota. This process results in the formation of short-chain fatty acids (SCFAs) and other organic acids that acidify the colonic environment,^2,21^ as well as gases such as H_2_, CO_2_, and CH_4_.^22,23^ Elevated colonic gas production leads to flatulence and increased intestinal wall tension by raising intraluminal pressures, triggering the perception of bloating, abdominal discomfort, and related symptoms via colonic mechanoreceptor simulation.^24,25^ The magnitude of symptoms is dependent on dietary fiber molecular size and structure. Larger, more complex fiber molecules, such as resistant starches, acacia gum, and arabinoxylans (AXs; a cereal derived fiber^26^), are fermented slower by fecal microbiota relative to inulin and resistant oligosaccharide molecules,^27–31^ which are less well tolerated.^32,33^ Accordingly, doses of resistant starch, resistant maltodextrin, and acacia gum of around 40 g/day are comparatively well tolerated.^19,34,35^ To our knowledge, effects of long-chain AXs on gastrointestinal (GI) symptoms have not been assessed beyond 15 g/day,^36^ and limited knowledge exists on how GI symptoms are linked to the gut microbiome.

Although symptoms can deter individuals from consuming fiber-rich foods, there is some evidence that suggests that humans can, at least to some degree, ‘adapt’ to sustained fiber consumption at high quantities, a process proposed to involve the gut microbiota.^37^ For instance, previous studies supplementing with acacia gum,^38^ partially hydrolyzed guar gum,^39^ inulin,^40^ resistant maltodextrin,^41^ and a blend of inulin and resistant maltodextrin^42^ have observed reductions in symptoms within two to four weeks of treatment. In addition, Mego and colleagues have shown that self-reported flatulence and number of gas evacuations decreased within two weeks of galactooligosaccharide treatment,^37^ with improvements stemming predominantly from reductions in the volume of intestinal gas produced.^43^ However, the dose of this study was, with 2.8 g/day, lower than what might be required for physiological and maximum bifidogenic effects.^44,45^ Therefore, whether humans can adapt to higher, more relevant supplementation doses and the factors that determine these responses (*e.g.*, the gut microbiota), remains insufficiently understood.

In previous studies, we have tested the effects of long-chain corn bran AX at daily doses of 25 and 35 grams (for women and men, respectively) on health^46^ and the gut microbiota^47^ using an exploratory randomized controlled trial (RCT) in adults with excess weight (BMI: 25–35 kg/m^2^). These studies revealed that AX exerted global changes on fecal bacterial community composition; promoted a range of bacterial taxa such as *Bifidobacterium longum*, *Blautia obeum*, *Subdoligranulum* sp., and *Prevotella copri*; increased fecal propionate concentrations (albeit highly individualized);^47^ and improved perceived satiety and measures of glucose homeostasis.^46^ In the current study, we extended our previous work in adults with excess weight to evaluate the severity of GI symptoms during high-dose AX supplementation, determined to what degree humans adapted to tolerate AX, and explored whether gut microbiota features or dietary-related factors associate with interpersonal differences in AX tolerance.

## Results

### AX induced moderate yet significant GI symptoms in comparison to non-fermentable microcrystalline cellulose (MCC)

**Figure 1.**
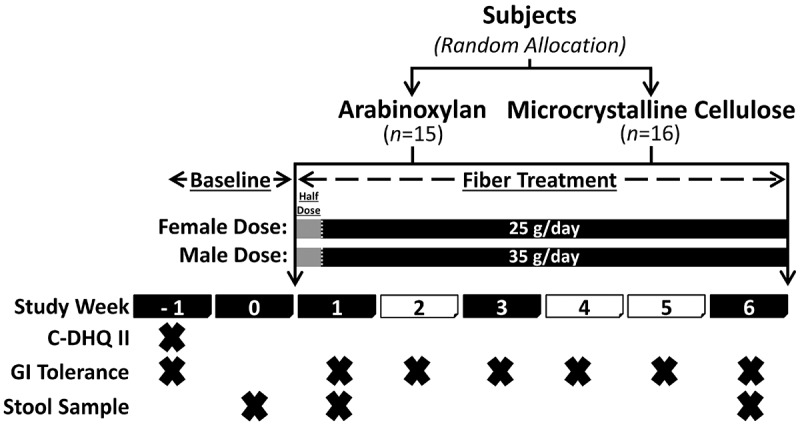
Study design.

**Figure 2.**
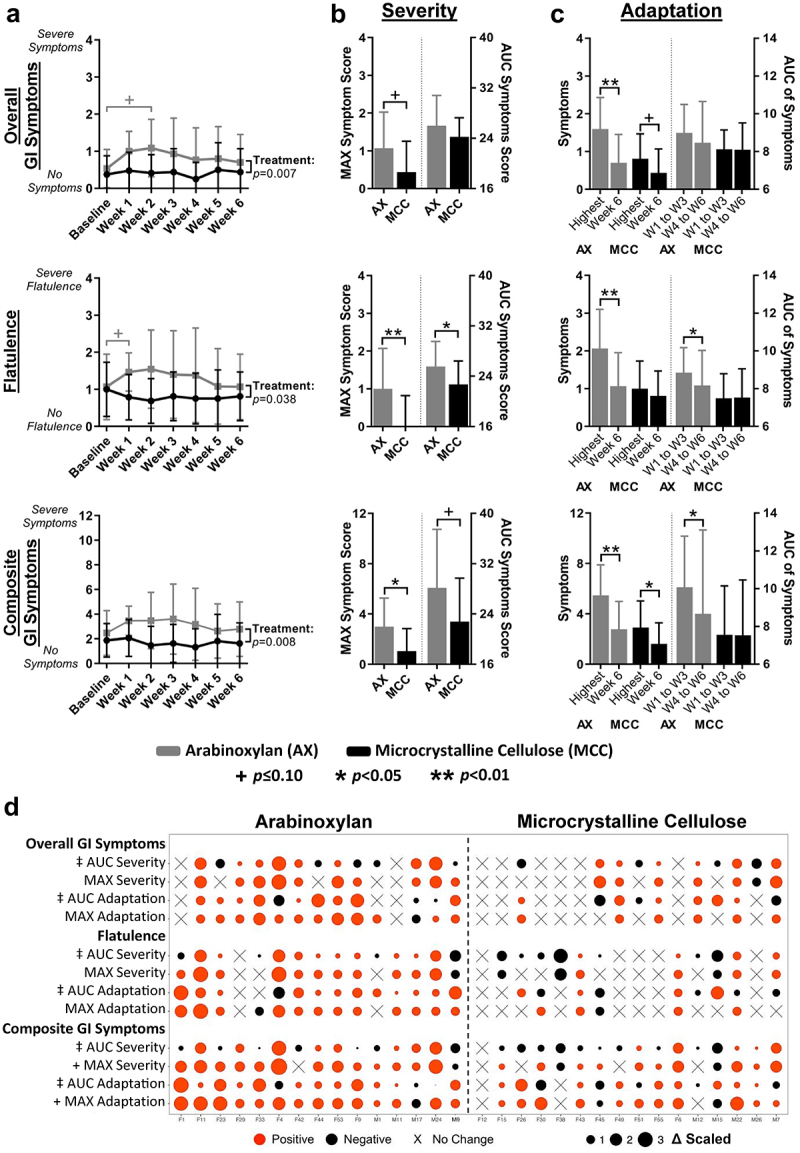
Symptoms in response to AX consumption and adaptation toward AX as compared to MCC consumption.

Thirty-eight volunteers with excess weight were enrolled in the study and instructed to supplement their diet, over six weeks, with either AX or MCC at a daily dose of 25 g (females) or 35 g (males); GI symptoms were reported at baseline, and weekly throughout the intervention (Figure 1 and Supplementary Table S1). Although AX was on average well tolerated (average symptom scores were < 2 points), overall symptoms, flatulence, bloating, stomach ache, and composite ratings were significantly higher when compared to subjects consuming MCC (treatment effect *p* < 0.05, generalized estimated equation models; Figure 2A and Supplementary Figure S1A). Considerable collinearity was detected between AX-induced symptoms; for instance, flatulence positively correlated with bloating (r_s_ = 0.55, *p* < 0.0001; Supplementary Figure S2). Comparison of maximum absolute change (MAX) severity scores (*i.e*., highest symptoms rating over entire treatment period) between AX and MCC treatments showed that flatulence, bloating, and composite ratings increased during AX consumption as compared to MCC (*p* < 0.05, Mann-Whitney tests; Figure 2B and Supplementary Figure S1B) (see Supplementary Figure S3A for explanation of severity scores). Accordingly, flatulence area under the curve severity (AUC_severity_) scores (*i.e.*, overall flatulence severity during the intervention) were higher for AX relative to MCC (*p* = 0.045), with differences in composite AUC_severity_ also approaching statistical significance (*p* < 0.1) (Figure 2B).

The ‘X’ indicates that the specific task was completed during the study week. C-DHQ II; Canadian Diet History Questionnaire II; GI, gastrointestinal.

(a) Overall symptoms, flatulence, and composite symptoms (sum of flatulence, bloating, and stomach aches) (see Supplementary Figure S1 for bloating and stomach ache data) during AX and MCC consumption. (b) MAX and AUC symptom scores for AX and MCC. (c) Highest symptom during weeks 1 to 5 and week 6 symptoms for AX and MCC, as well as the AUC of symptoms from weeks 1 to 3 and weeks 4 to 6 (see Supplementary Figure S3 for explanation of scores). Data in (a) were analyzed using generalized estimated equation models with Bonferroni corrections, in (b) using Mann-Whitney tests, and in (c) using Wilcoxon tests with Bonferroni corrections. Data reported as mean ± SD. Statistical significance considered at *p* < 0.05. (d) Individualized symptom severity and adaptation scores during AX and MCC consumption. Red circles represent an increase, black circles represent a decrease, and ‘X’ represents no change in the feature during the intervention. The circle size is proportional to the scaled magnitude change relative to baseline or week 6. ‡ Feature scaled by (AUC_severity_–24/SD) or (AUC_adaptation_–1/SD), + Feature scaled by (MAX/SD). AUC, area under the curve; GI, gastrointestinal.

### Subjects showed an ‘adaptation’ to AX in that symptoms declined over time

Symptoms ratings for flatulence, bloating, stomach ache, and composite scores improved during AX consumption, with ratings at week 6 being lower than MAX values and often close to baseline values (*p* < 0.05, Wilcoxon tests; Figure 2C and Supplementary Figure S1C) (see Supplementary Figure S3B for explanation of adaptation scores). Although minor and unrelated to microbial fermentation, significant improvements in composite ratings were also detected at six weeks of MCC consumption relative to MAX composite values (*p* = 0.01; Figure 2C). In addition to MAX shifts, AUCs of weeks 1 to 3 were significantly higher than those of weeks 4 to 6 for both flatulence (*p* = 0.02) and composite scores (*p* = 0.02) during AX but not MCC consumption (Figure 2C). These findings suggest that although corn bran AX, at doses of 25 g/day and 35 g/day, induced symptoms and primarily flatulence, effects were temporary, as most subjects ‘adapted’ to AX within six weeks of sustained consumption.

### Inter-individualized variation in symptoms is linked to fecal microbiota composition

Although significant increases in GI symptoms were detected in the study cohort, symptom severity and the degree of adaptation were highly individualized (Figure 2D and Supplementary Figure S1D). MAX composite severity scores were increased by ≤ 2 out of 12 points for 40% of subjects, while 33% reported scores ≥ 4. For most individuals (80%), composite ratings reverted to baseline, as MAX composite severity and adaptation scores were equivalent (*i.e.*, difference of ≤ 1 point). However, for the two subjects that reported the most intense symptoms, symptoms did not recover completely (*i.e.*, F4 reduced from 10 to 6 points; M24 reduced from 9 to 6 points).

Symptoms linked to increased fiber consumption such as flatulence and bloating are likely the result of gas formation during fermentation by the gut microbiota,^22,23^ which shows substantial inter-individual variation.^48–50^ We therefore performed a systematic analysis between the AUC_severity_ of GI symptoms during AX and MCC supplementation and the relative abundance of bacterial taxa at different taxonomic levels. Spearman’s correlation analyses also included co-abundance response groups (CARGs), which are groups of inter-correlated operational taxonomic units (OTUs),^47^ since bacteria collaborate during fiber fermentation and engage in complex cross-feeding interactions within what can be considered ecological guilds.^51^ As fecal samples were collected in weeks 1 and 6, AUC_severity_ ratings from weeks 1 to 3 were correlated with measurements in week 1 samples, and AUC_severity_ ratings from weeks 4 to 6 were correlated with measurements in week 6 samples.

**Figure 3.**
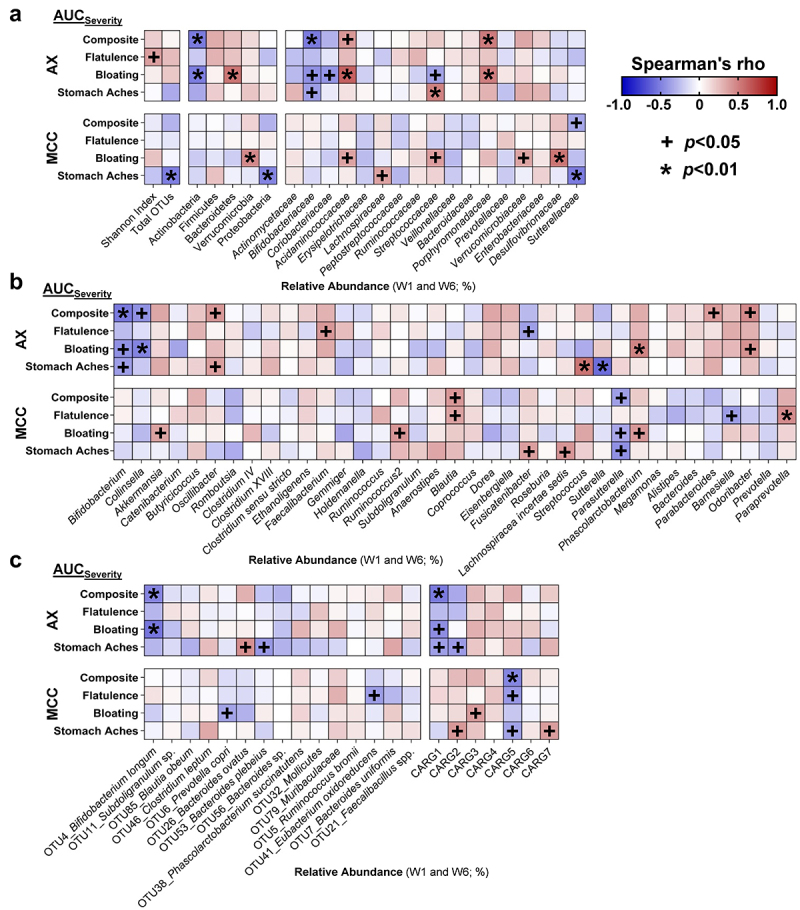
Associations between symptom severity and bacterial abundance during AX and MCC consumption.

This analysis showed that neither α-diversity, assessed by Shannon index, nor the total number of OTUs was associated with severity ratings for AX (*p>*0.01; Figure 3A). The analysis at phylum-level revealed that, during AX consumption, the abundance of Actinobacteria negatively correlated with composite (r_s_ = -0.53, *p* = 0.003) and bloating (r_s_ = -0.53, *p* = 0.003) severity, while Bacteroidetes positively correlated with bloating severity (r_s_ = 0.53, *p* = 0.002) (Figure 3A). At lower taxonomic levels, the family *Bifidobacteriaceae* (r_s_ = -0.52, *p* = 0.003; Figure 3A) and genus *Bifidobacterium* (r_s_ = -0.52, *p* = 0.003; Figure 3B) negatively correlated with composite severity, while negative correlations with both bloating and stomach ache severity approached statistical significance (*p* < 0.05). In contrast, positive correlations were detected between the family *Porphyromonadaceae* and composite (r_s_ = 0.52, *p* = 0.003) and bloating (r_s_ = 0.50, *p* = 0.005) severity; with *Oscillibacter*, *Parabacteroides*, and *Odoribacter* genera also showing positive correlations with composite severity that approached statistical significance (*p* < 0.05).

Heatmaps show Spearman’s correlations between the composite, flatulence, bloating, and stomachache AUC_severity_ scores (segmented into weeks 1 to 3 and weeks 4 to 6) and the all bacterial (a) phyla, families, (b) genera, and (c) CARGs with average relative abundances above 0.15%, and (c) AX-responsive OTUs (weeks 1 and 6). Statistical significance considered at *p* < 0.01. AUC, area under the curve; AX, arabinoxylan; CARG, co-abundance response group; MCC, microcrystalline cellulose; OTU, operational taxonomic unit.

For OTUs and CARGs, analyses were focused on only AX-responsive OTUs (termed ‘significant OTUs’^47^) to avoid type-1 error, while all seven CARGs were included. This analysis revealed that the relative abundance of *B. longum* (OTU4) during AX consumption negatively correlated with composite (r_s_ = -0.48, *p* = 0.007) and bloating (r_s_ = -0.54, *p* = 0.002) severity (Figure 3C). The relative abundance of CARG1 – the CARG dominated by *B. longum*^47^ (Supplementary Figure S4) – was also shown to negatively correlate with composite severity (r_s_ = -0.53, *p* = 0.003), while negative correlations for both bloating and stomach ache severity approached statistical significance (*p* < 0.05). The above-mentioned correlations were not detected during MCC consumption (*p* > 0.1; Figure 3), which might indicate that these associations are related to symptoms induced by AX fermentation. Overall, these findings suggest that higher abundance of *B. longum* during AX supplementation designates better GI tolerance, while higher *Porphyromonadaceae* designates worse GI tolerance.

### Severity and adaptation scores correlate with baseline and AX-Induced shifts in microbiota composition

**Figure 4.**
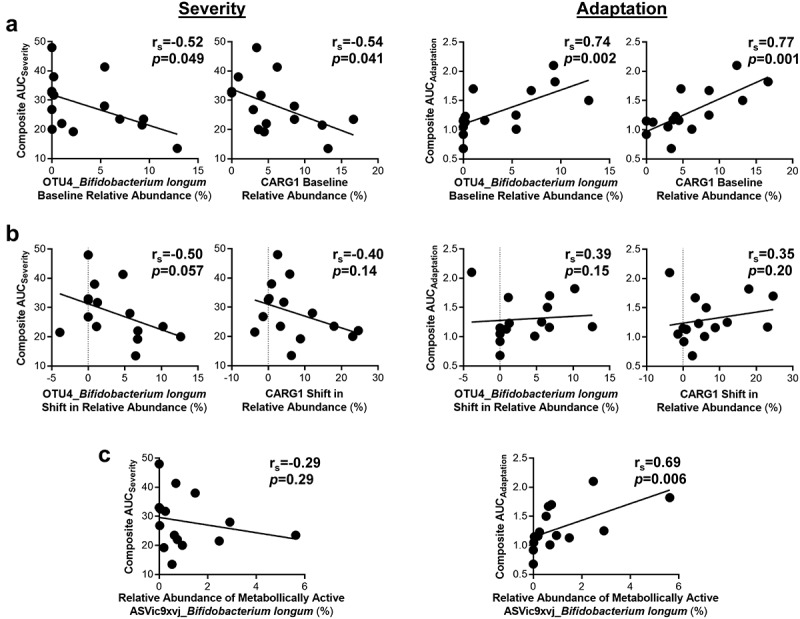
Improved composite scores during AX consumption were linked to the relative abundance of Bifidobacterium longum.

To evaluate if the tolerance of AX is pre-determined by the baseline microbiota, we assessed whether MAX and AUC severity and adaptation scores associated with relative pre-treatment abundances of the significant OTUs and CARGs. This analysis revealed that greater relative abundances of *B. longum* (OTU4; r_s_ = -0.67, *p* = 0.007) and CARG1 (r_s_ = -0.67, *p* = 0.008) prior to AX supplementation associated with lower bloating AUC_severity_ scores (Supplementary Figure S5), with composite AUC_severity_ also showing a tendency to be lower (*p* < 0.05; Figure 4A). Relative pre-treatment abundances of *B. longum* (OTU4; r_s_ = 0.74, *p* = 0.002) and CARG1 (r_s_ = 0.77, *p* = 0.001) were also positively associated with better composite AUC_adaptation_ scores.

Spearman’s correlations between *B. longum*, CARG1 (CARG dominated by *B. longum*), and AX-induced composite AUC_severity_ (left) and AUC_adaptation_ (right) scores. Correlations were performed on (a) baseline or (b) shifts in the relative fecal abundance of *B. longum* (OTU4) and CARG1, or (c) the relative *ex vivo* abundance of *B. longum* (ASVic9×vj). For correlations with flatulence, bloating, and stomachache scores see Supplementary Figures S5, S6, and S7. The best-fitting line is the linear regression line. Statistical significance considered at *p* < 0.01. ASV, amplicon sequence variant; AUC, area under the curve; AX, arabinoxylan; CARG, co-abundance response group; OTU, operational taxonomic unit.

Next, we determined whether AX-induced shifts (week 6 – baseline) of significant OTUs and CARGs correlated with severity and adaptation scores. While associations were not detected for *B. longum* (OTU4) or CARG1 (*p* > 0.05; Figure 4B), enrichment of *Subdoligranulum* sp. (OTU11) by AX was associated with higher composite AUC_adaptation_ scores (r_s_ = 0.68, *p* = 0.007), with bloating and stomach ache AUC_adaptation_ scores also showing a tendency to be higher (*p* < 0.05) (Supplementary Figure S6).

Finally, we applied bioorthogonal non-canonical amino acid-tagging (BONCAT) to identify the bacterial consortia within the fecal microbiota of the subjects that are involved in the fermentation of AX,^46^ and then assessed associations between the activated bacterial amplicon sequence variants (ASVs) and MAX and AUC severity and adaptation scores. This analysis revealed that only the relative abundance of *B. longum* (ASVic9×vj), within the active consortia, positively associated with composite (r_s_ = 0.69, *p* = 0.006; Figure 4C) and bloating (r_s_ = 0.68, *p* = 0.006; Supplementary Figure S7) AUC_adaptation_ scores. Taken together, the findings suggest that the adaptation to tolerate AX at high-doses is influenced more by the relative abundances of specific AX degrading microbes such as *B. longum*, than by AX-induced changes in community membership.

### Tolerance to AX is associated with changes in fecal pH and SCFAs

**Figure 5.**
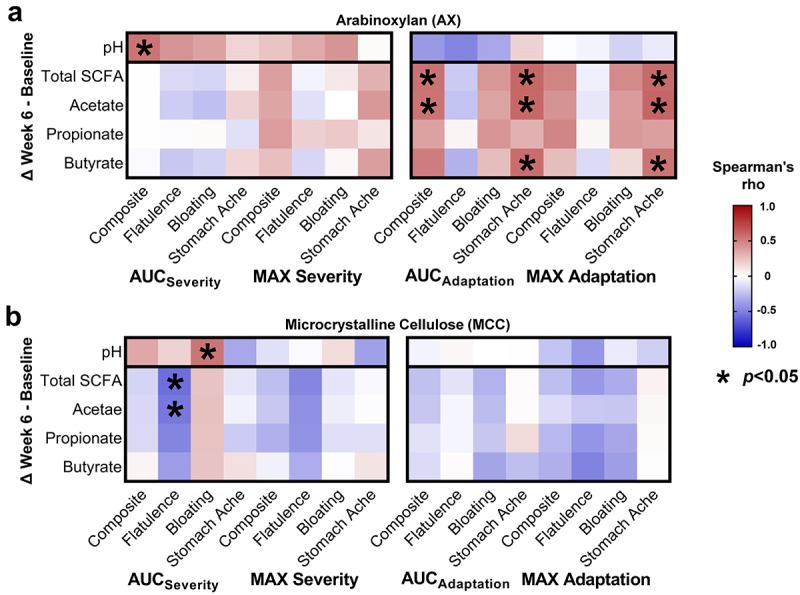
Shifts in fecal pH and SCFA concentrations correlated with the severity and adaptation of AX- and MCC-induced symptom.

Previously, we showed that AX increased fecal propionate (overall effect *p* = 0.015, Friedman’s test), while remaining SCFAs and fecal pH did not change (overall effect *p* > 0.1).^47^ Given the substantial variation in these parameters, we asked whether inter-subject differences in severity and adaptation were linked to different shifts in fecal pH (week 6 – baseline). This analysis showed that fecal acidification was associated with both lower composite AUC_severity_ for AX (r_s_ = 0.54, *p* = 0.039; Figure 5A) and bloating AUC_severity_ for MCC (r_s_ = 0.54, *p* = 0.034; Figure 5B). Fecal acidification was also associated with greater abundances of *B. longum* (OTU4) during AX consumption (r_s_ = -0.44, *p* = 0.016; Supplementary Figure S8A), a correlation that approached statistical significance for MCC (r_s_=-0.30, *p* = 0.093; Supplementary Figure S8B).

Heatmaps show Spearman’s correlations between fecal pH and SCFA shifts (week 6 – baseline) and the MAX and AUC severity and adaptation scores for (a) AX and (b) MCC. Statistical significance considered at *p* < 0.05. AUC, area under the curve; AX, arabinoxylan; MCC, microcrystalline cellulose; SCFA, short-chain fatty acid.

Analysis of fecal SCFA shifts further revealed positive correlations between composite AUC_adaptation_ scores and total SCFAs (r_s_ = 0.55, *p* = 0.036), and acetate (r_s_ = 0.54, *p* = 0.039) (Figure 5A). Stomach ache AUC_adaptation_ scores were also correlated positively with total SCFAs (r_s_ = 0.60, *p* = 0.021), acetate (r_s_ = 0.59, *p* = 0.022), and butyrate (r_s_ = 0.57, *p* = 0.027). Although no associations were detected between pH and SCFA shifts (*p* > 0.1, data not shown), positive correlations that approached statistical significance were detected between acetate shifts and *B. longum* (OTU4) abundance during AX (r_s_ = 0.33, *p* = 0.072; Supplementary Figure S8A) and MCC (r_s_ = 0.30, *p* = 0.097; Supplementary Figure S8B) consumption. Overall, these findings suggest that the adaptation to tolerate AX is partially driven by inter-individual differences in microbial fermentation of AX and subsequent acidification of the colonic environment, primarily through acetate (the major SCFA produced by bifidobacteria).^52^

### Association between baseline diet and GI tolerance of AX and MCC

Previous research has shown that habitual intake of animal- vs. plant-based diets differentially affect gut microbiota composition and metabolic activity.^6–55^ We therefore investigated whether pre-treatment, calorie-adjusted intake of meat/meat alternatives, whole grains (where AX is the dominant fiber^26^), cholesterol (found only in animal-based foods), and dietary fiber, or the ratio between animal- and plant-based foods/nutrients were linked to MAX and AUC severity and adaptation scores.

**Figure 6.**
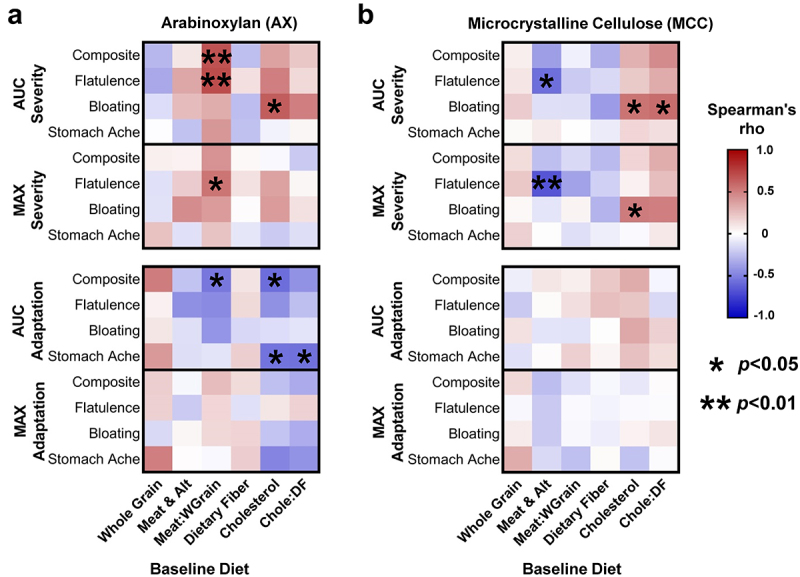
Baseline diet history correlated with symptom severity and adaptation during AX and MCC consumption.

This analysis revealed that, for AX, a higher proportion of meat/meat alternatives to whole grains in the subjects’ pre-treatment diet correlated positively with composite (r_s_ = 0.68, *p* = 0.007) and flatulence (r_s_ = 0.70, *p* = 0.005) AUC_severity_ scores, and negatively with composite AUC_adaptation_ (r_s_ = -0.54, *p* = 0.042) (Figure 6A). The ratio of meat/meat alternatives to whole grains was further shown to positively correlate with MAX flatulence severity (r_s_ = 0.54, *p* = 0.04). Interestingly, for MCC, the calorie-adjusted intake of meat/meat alternatives also correlated inversely with flatulence MAX (r_s_ = -0.67, *p* = 0.005) and AUC (r_s_=-0.57, *p* = 0.02) severity scores (Figure 6B). In terms of nutrients, higher intakes of cholesterol correlated positively with bloating AUC_severity_ for AX (r_s_ = 0.63, *p* = 0.013) and MCC (r_s_ = 0.54, *p* = 0.03). Negative associations were also observed between cholesterol intake and composite (r_s_ = -0.58, *p* = 0.027) and stomach ache (r_s_ = -0.55, *p* = 0.036) AUC_adaptation_ scores for AX but not MCC (Figure 6). Although no associations were detected between intakes of whole grains or meat/meat alternatives and the relative abundance of *B. longum* (OTU4) at baseline (*p* > 0.1), an inverse correlation that approached statistical significance was detected between intakes of cholesterol and baseline *B. longum* levels (r_s_ = -0.35, *p* = 0.052; Supplementary Figure S9). In summary, habitual intake of more animal-based foods and less whole grains appears to elevate the perceived severity of GI symptoms and lessen symptom improvements during AX supplementation.

Heatmaps show Spearman’s correlations between calorie-adjusted intakes of animal- and plant-based foods/nutrients at baseline, and the MAX and AUC severity and adaptation scores for (a) AX and (b) MCC. Statistical significance considered at *p* < 0.05. chole:DF, ratio of dietary cholesterol to dietary fiber; meat:wgrain, ratio of meat/meat alternatives to whole grains. AUC, area under the curve; AX, arabinoxylan; MCC, microcrystalline cellulose.

## Discussion

In agreement with findings from human trials with other fermentable fibers,^24,32,33^ consumption of corn bran AX at high doses of 25 g/day (females) or 35 g/day (males) intensified GI symptoms. After higher symptoms within the first three weeks of supplementation, ratings reverting almost completely back to baseline levels during weeks 4 to 6. This observation is relevant as it indicates that humans can adapt to high amounts of AX within a relatively short time frame. Severity and adaptation responses were both subject-dependent and correlated with gut microbiota composition, fecal acidification, and baseline diet. These correlations provide potential explanations for the inter-individualized variation in the adaptations toward tolerating dietary fibers, which can serve as a basis for the development of personalized microbiome-targeted or dietary strategies aimed to increase fiber consumption by alleviating fiber-induced symptoms.

Compared to many other fibers, AX is rather well tolerated. Inulin and resistant oligosaccharides appeared to induce symptoms at doses of ~10 g/day that are equivalent or higher than what we detected for 25–35 g/day of corn bran AX.^32,33,45,56^ Higher tolerance of corn bran AX might arise from its complex molecular structure.^47^ Accordingly, *in vitro* fecal fermentation studies have demonstrated lower gas production rates by corn bran AX and hydrolyzates thereof relative to fructooligosaccharides and even AXs with more simple molecular structures (*i.e.*, sorghum and rice AX).^27,29,31^ Other complex fibers characterized by slow fermentation rates *in vitro* (*e.g.*, resistant starch, acacia gum, and polydextrose^28,30,57^) have also shown to be more tolerable at doses above 30 g/day.^18, 31 − 33,47^ Overall, the findings suggest that increased structural complexity attenuates the rate of fiber fermentation, which permits colonic absorption and evacuation of gases without colonic buildup, leading to improved tolerance.^23^

Previous studies have suggested that symptoms of fiber consumption are predominantly influenced by the gut microbial community, affecting colonic gas production and the removal of H_2_.^37,43,58^ While associations were not detected with putative hydrogenotrophic bacterial taxa in our study (*i.e.*, acetogens, methanogens, and sulfate-reducing bacteria), *Bifidobacterium* abundance correlated with severity and adaptation of both bloating and composite symptoms. These correlations are in agreement with previous findings from a longitudinal study in healthy individuals, where higher *Bifidobacterium* abundance was inversely associated with abdominal pain and intestinal discomfort.^59^ In addition, probiotic intervention trials showed that *Bifidobacterium* strains reduced bloating in irritable bowel syndrome.^60–62^ Although cause and effect relationships and mechanisms cannot be established in our study, several aspects of the metabolism of *Bifidobacterium* provide a potential explanation for reduced gas production rates during AX fermentation. First, *Bifidobacterium* species are non-gas-producing.^52^ Competition of *B. longum* for AX could therefore reduce net colonic gas production by other organisms. Second, *Bifidobacterium* produce lactate and acetate from carbohydrate fermentation,^52^ which acidify the colonic environment. In general, acidification has been shown to lower the rate and net production of H_2_ during microbial fermentation of carbohydrates.^63^ This mechanism is supported by the correlations between GI tolerance to AX and shifts in fecal pH and acetate (the dominant SCFA produced by bifidobacteria) in our study. Interestingly, associations were also detected with both *ex vivo* activity of AX-utilizing *B. longum* and CARG1, the dominate responding co-abundance cluster during AX consumption that encompassed six inter-correlated AX-responding OTUs, including *B. longum* and *Subdoligranulum* sp., which also produces lactate.^47^,^64^ Overall, correlations detected in our study suggest that severity and adaptation of AX-induced symptoms are to some degree determined by the bacterial consortia involved in AX degradation and specifically *B. longum’s* position within the active consortia.

We can only speculate about the mechanisms by which adaptation to AX occurs. One possibility is that bacteria within CARG1, for instance *B. longum*, adapt to become more efficient at utilizing AX. Cooperative cross-feeding interactions among members of CARG1, such as between *B. longum* (proposed primary degrader) and *B. obeum* or *Subdoligranulum* sp. (proposed secondary fermenters),^47^ may also become more efficient. Previously, we have shown that while AX-induced shifts in gut microbiota composition manifested within one week without further changes, adaptation in the production of propionate was detected in 40% of subjects and predicted by CARG1 shifts.^47^ Therefore, it is feasible that within six weeks, this bacterial consortium exhibits a functional adaptation toward reduced gas production during AX fermentation, improving symptoms perceived by the individual. However, other mechanisms are also possible; for instance, *B. longum* could mitigate the perception of visceral stimuli through upregulation of neurotransmitters, which would improve symptoms without altering gas production.^65^ Therefore, future studies are needed that apply more sophisticated techniques – such as whole metagenomic, shotgun sequencing in combination with ingestible gas-sensing capsules^22^ – to evaluate adaptation.

Our correlation analyses between GI symptoms and diet indicate that habitual consumption of a diet higher in whole grains at the expense of animal-based foods enhances the tolerance of AX. Previous studies have suggested that long-term dietary habits play a role in shaping the composition and metabolic activity of the gut microbiota.^53,55,66^ For instance, higher intakes of animal proteins and fats have been linked to greater relative abundances of the family *Porphyromonadaceae* and genera *Odoribacter*, *Parabacteroides*, and *Bacteroides*.^53^ It is possible that dietary patterns rich in whole grains (which are rich in AXs) over time select for microbes that can adapt to more efficiently ferment these substrates, which might be linked to less H_2_ production during AX fermentation and explain the inter-individualized adaptations observed toward the tolerance of AX. Diets rich in animal products, on the other hand, might select for microbes that are less AX-adapted and produce more H_2_. These speculations would align with the inverse association between *B. longum* and dietary cholesterol that approached significance and the positive correlation observed between *Porphyromonadaceae* and composite AUC_severity_, with positive correlations for *Odoribacter* and *Parabacteroides* approaching significance. Other groups have additionally reported positive associations between the genera *Parabacteroides* and *Bacteroides* and increased flatulence.^67,68^ Overall, these findings provide further support that the gut microbiota can adapt toward dietary fiber to attenuate GI symptoms and suggest that habitual diet might be an important factor in this process that contributes to personalized tolerance of fibers.

The findings obtained in this study are important for several reasons. First, our study showed that humans are capable of adapting to dietary fiber doses that modulate microbiota community composition and function (propionate)^47^ and show clinical effects (satiety and insulin resistance).^46^ Adaption appears to occur both short-term (during the AX intervention) and longer-term (through the habitual diet) and constitutes an avenue by which tolerance to physiologically relevant and perhaps even ancestral doses can be both achieved, making closing the fiber gap feasible. Second, even though the design of this study does not allow causal inferences between symptom adaptations and the gut microbiota, the associations observed, specifically with the abundance of *B. longum* and fecal acetate (the dominant SCFA produced by *B. longum*), point toward possible mechanisms by which tolerance arises. Further research is needed to elucidate the mechanisms that underly personalized adaptations toward the tolerance of physiologically relevant levels of fiber consumption. Third, this information provides a basis to use *B. longum* strains (and perhaps other members of CARG1) well adapted to competitively utilize AX as probiotics to enhance the tolerance of AX. However, a rigorously designed RCT would be needed to confirm whether this synergistic synbiotic strategy attenuates GI symptoms induced by AX, especially in those individuals experiencing more sever symptoms. Such an RCT would aid in inferring causality between GI tolerance and the gut microbiota. Finally, our findings suggest that microbiome benefits are adaptable and can therefore be altered through selection, opening options for evolutionary-based strategies to modulate the gut microbiome.

## Materials and methods

### Registration

As described previously,^46,47^ this six-week, exploratory RCT was registered with ClinicalTrials.gov, registry number NCT02322112, as part of a large four-arm RCT aimed to compare the effects of AX, acacia gum, resistant starch type-IV, and MCC consumption on the gut microbiota and human health. In response to requests by reviewers of a grant application, the AX arm was separated from the original RCT and data from the 15 protocol completers were analyzed independently and compared to data from the first 16 completers of the MCC protocol (non-fermentable controls). Study procedures were approved by the University of Alberta Health Research Ethics Board, identifier Pro00050274, with written informed consent obtained prior to participant enrollment (for study procedures refer to Ref.^47^).

### Study design and subjects

Thirty-eight volunteers with excess weight were enrolled in the study and instructed to supplement their diet, over six weeks, with either AX or MCC at a daily dose of 25 g (females) or 35 g (males) (Figure 1). AX was AGRIFIBER SFC (previously named BIOFIBER GUM), a fermentable long-chain AX isolated from corn bran (AgriFiber Solutions LLC, Illinois, USA), while the non-fermentable control was MICROCEL MC-12, a large particle wood derived MCC (Blanver Farmoquimica LTDA, São Paulo, Brazil).^47^ Thirty-one subjects aged 33 ± 9 years and body mass index 28.7 ± 2.3 kg/m^2^ completed the intervention and were analyzed per-protocol, which consisted of 21 females and 10 males (AX arm: 10F and 5 M; MCC arm: 11F and 5 M; Supplementary Table S1). On average, the total intake of dietary fiber was increased during the intervention from 19 ± 5 and 21 ± 11 g/day to 40 ± 5 and 56 ± 10 g/day for females and males, respectively (assessed by two 24-hr recalls).^46^

### Assessment of habitual diet at baseline

Diet history was assessed at baseline using the online one-month Canadian Diet History Questionnaire II (C-DHQ II), a food frequency questionnaire adapted for Canada.^69^ C-DHQ II responses were analyses using Diet*Calc software (v1.5.0) and an updated C-DHQ II nutrient database, which included eight additional food group variables that align with Canada’s 2007 Food Guide serving-size-equivalents.^70^ Prior to statistical integration with GI symptoms, C-DHQ II data were adjusted for total caloric intake.^71^

### Assessment of perceived GI symptoms

Participants reported GI symptoms at baseline, and weekly throughout the intervention by completing a symptoms diary. At the end of each week, subjects rated their overall symptoms, flatulence, bloating, and stomachache intensity using a scale from 0 (no symptoms) to 4 (severe symptoms).^48^ A composite symptom rating was calculated by summing flatulence, bloating, and stomachache ratings, resulting in a possible range from 0 to 12 (higher ratings corresponded to more severe symptoms).

Two different approaches were used to quantify the “severity” of GI symptoms in response to AX and the degree of “adaptation” for each subject: maximum absolute change (MAX) in symptoms throughout the intervention and area under the curve (AUC) of symptoms (explained in Supplementary Figure S3). Although alike, MAX was used to ascertain extreme changes in symptom intensity, while AUC averaged the change in symptoms during the intervention.

To assess severity, the MAX severity score was calculated by subtracting baseline ratings from the highest reported rating during weeks 1 to 5, where higher scores represent more intense symptoms. To calculate the AUC_severity_ score, AUCs from weeks 1 to 6 were computed, where higher scores correspond to more severe symptoms during the six-week intervention relative to baseline. To evaluate adaptation, the MAX adaptation score was calculated by subtracting week 6 ratings from the highest reported rating during weeks 1 to 5. While higher scores represent greater reductions in symptom intensity, differences between the ratings indicate the degree to which symptoms adapted. To determine AUC_adaptation_, weeks 1 to 3 and weeks 4 to 6 AUCs were first computed, wherein differences between the AUCs indicate the degree of adaptation. AUC_adaptation_ scores were then calculated by dividing the AUC from weeks 1 to 3 by the AUC from weeks 4 to 6, where higher scores equate to better adaptation during the final three weeks of treatment.

### Fecal microbiota compositional and functional analyses

Compositional and functional features of the fecal microbial community were assessed at baseline and in response to one and six weeks of AX consumption. Findings from 16S rRNA gene amplicon profiling of fecal microbiota, including the identification of CARGs (groups of inter-correlated OTUs), as well as the characterization of fecal pH and SCFAs have been published previously by Nguyen et al.^47^

To confirm whether bacterial taxa within the fecal microbial community specifically involved in the fermentation of AX influenced the severity and adaptation of GI symptoms during AX consumption, we utilized an *ex vivo* approach based on BONCAT.^46,72^ Briefly, this approach fluorescently labeled bacterial cells that were metabolically active during a 6-hr anaerobic incubation with AX, and then active bacteria were isolated using fluorescence-activated cell sorting. Next, 16S rRNA gene amplicon sequencing and bioinformatic analyses based on ASVs were used to identify the precise bacterial consortium within each participant’s fecal microbiota that contributed to AX fermentation. Initial findings, as published previously by Deehan et al,^46^ suggest that the fermentation of AX involved numerous members of the fecal bacterial community (*i.e.*, around 69% of observed ASVs), which included multiple *Bacteroides* spp., *Blautia* spp., and *B. longum* as part of the most abundant bacterial ASVs (average relative abundance > 1.0%; Supplementary Table S2). 16S rRNA gene amplicon data are available for download at the NCBI Sequence Read Archive under BioProjects: PRJNA564636 (fecal) and PRJNA630848 (*ex vivo*).

### Statistical analyses

Generalized estimated equation models with Bonferroni post-hoc tests were applied to GI symptom and composite ratings to determine differences between-groups and within-group differences relative to baseline. Differences between AX and MCC for the calculated MAX severity and AUC_severity_ scores were determined by Mann-Whitney tests. To test for adaption to AX and MCC, Bonferroni corrected Wilcoxon tests were applied to determine differences between the highest rating reported during weeks 1 to 5 and the week 6 rating, as well as the AUC of weeks 1 to 3 ratings and weeks 4 to 6 ratings.

To determine whether compositional and functional features of the fecal microbiota or dietary factors correlated with symptom severity or adaptation, Spearman’s correlations were applied. Significant associations were first identified by correlating flatulence, bloating, stomachache, and composite severity scores with microbiota compositional variables measured in fecal samples during the intervention. As fecal samples were collected in weeks 1 and 6, symptoms during weeks 1 to 3 were hereby correlated with measurements in week 1 fecal samples, and symptoms during weeks 4 to 6 were correlated with measurements in week 6 fecal samples. For these analyses, all bacterial phyla, families, genera, and CARGs with average relative abundances above 0.15% (considering all fecal samples) were systematically assessed, while only OTUs significantly affected by AX (henceforth referred to as ‘significant OTUs’) were considered to reduce the chance of type I error from multiple comparisons (15 OTUs instead of 100; Supplementary Table S2). Significant OTUs and CARGs were evaluated further to determine if their relative abundance at baseline correlated with MAX and AUC severity and adaptation scores. AX-induced shifts (week 6 – baseline) of significant OTUs, CARGs, pH, and SCFAs were correlated with MAX and AUC severity and adaptation scores to assess whether microbial responses to AX relate to symptoms. Finally, to elucidate whether tolerance to AX is linked to bacterial taxa shown to be involved in the fermentation of AX, correlations were assessed between MAX and AUC severity and adaptation scores and the most abundant bacterial ASVs (average relative abundance > 1.0%; 22 ASVs instead of 90; Supplementary Table S2) within the active bacterial consortia as established by BONCAT.

Given the connections between habitual intake of animal- and plant-based diets and gut microbiome composition^73^ and that AXs constitute the main non-cellulose fiber in cereal grains,^24^ we further investigated whether calorie-adjusted intakes – during the month prior to treatment – of meat/meat alternatives (included eggs, legumes, nuts, and seeds), cholesterol (a nutrient found only in animal-based foods), whole grains, and dietary fiber, or the ratio between these food groups or nutrients, correlated with MAX and AUC severity and adaptation scores. All statistical analyses were performed using GraphPad Prism v8.4.3, except from generalized estimated equation models, which were performed using the statistical software R v3.5.3. Statistical significance was considered at *p* < 0.01 for correlations with microbiota compositional data (to account for multiple comparisons), and at *p* < 0.05 for the remaining analyses.

## Supplementary Material

Supplemental Material

## Data Availability

16S rRNA gene amplicon sequencing data are publicly available in the NCBI Sequence Read Archive and can be accessed through BioProject accession numbers PRJNA564636 (fecal) and PRJNA630848 (*ex vivo*).
