# Supplementary material for: Adaptation to tolerate high doses of arabinoxylan is associated with fecal levels of Bifidobacterium longum
Source: Gut Microbes. 2024 Jun 11;16(1):2363021. doi: 10.1080/19490976.2024.2363021 (PMC11174067; doi:10.1080/19490976.2024.2363021)
Supplement: Supplemental Material [file KGMI_A_2363021_SM0050.pdf]

Supporting Information

Adaptation to tolerate high doses of arabinoxylan is associated with fecal levels of *Bifidobacterium longum*

Edward C. Deehan, Zhengxiao Zhang, Nguyen K. Nguyen, Maria Elisa Perez-Muñoz, Janis Cole, Alessandra Riva, David Berry, Carla M. Prado, and Jens Walter

Table of Contents

Table S1. Baseline subject characteristics

Table S2. Relative abundance of bacterial taxa affected by AX as assessed 16S rRNA gene amplicon sequencing of the fecal and *ex vivo* microbiota.

Figure S1. Characterizing the severity and adaptation of bloating and stomach ache symptoms during AX and MCC consumption.

Figure S2. Multicollinearity detected between treatment-induced changes in assessed gastrointestinal symptoms.

Figure S3. Graphical representation of how severity and adaptation scores were determined.

Figure S4. Dominant CARG enriched during AX consumption.

Figure S5. Associations between severity and adaptation scores and pre-treatment abundances of AX-responsive bacterial taxa in fecal samples.

Figure S6. Severity and adaptation scores correlated with the shifts in AX-responsive bacterial taxa in fecal samples.

Figure S7. Associations between severity and adaptation scores and the bacterial taxa that utilise AX *ex vivo*.

Figure S8. Associations between *Bifidobacterium longum* abundance and fecal pH and acetate shifts during AX and MCC consumption.

Figure S9. Associations between baseline Bifidobacterium longum abundance and baseline diet history.

Table S1. Baseline subject characteristics.

	AX Arm	MCC Arm	*p* value
Number	15	16	
Sex (female/male)	10/5	11/5	
Age (y)	33.7 ± 9.7	32.1 ± 7.4	0.91
Height (cm)	171.5 ± 8.4	168.8 ± 7.6	0.25
Weight (kg)	84.8 ± 12.3	81.9 ± 10.5	0.40
Body mass index (kg/m^2)	28.7 ± 2.7	28.7 ± 2.0	0.99
Waist circumference (cm)	95.7 ± 8.8	92.8 ± 6.2	0.30
Percent body fat (%)	33.0 ± 9.3	32.0 ± 7.3	0.63
Females	36.4 ± 2.9	38.0 ± 6.1	0.20
Males	22.5 ± 3.6	23.0 ± 5.3	0.84

Data presented as mean ± SD and analyzed by Mann-Whitney tests.

Table S2. Relative abundance of bacterial taxa affected by AX as assessed 16S rRNA gene amplicon sequencing of the fecal and *ex vivo* microbiota.

Fecal Analysis[1]			*Ex Vivo* Analysis[2]		
**Bacterial OTU**	**Classification**	**Relative Abundance**	**Bacterial ASV**	**Classification**	**Relative Abundance**
4	*Bifidobacterium longum*	7.04 ± 6.76	ic9xvj	*Bifidobacterium longum*	1.11 ± 1.53
6	*Prevotella copri*	3.63 ± 6.76	935uue	*Bacteroides cellulosilyticus*	3.74 ± 10.78
11	*Subdoligranulum* sp.	2.41 ± 3.27	hiw9kp	*Bacteroides dorei*	1.03 ± 1.61
85	*Blautia obeum*	1.33 ± 1.30	5mi71s	*Bacteroides koreensis*	1.88 ± 4.17
56	*Bacteroides* sp.	0.84 ± 3.04	352ie0	*Bacteroides koreensis*	1.25 ± 2.40
38	*Phascolarctobacterium succinatutens*	0.78 ± 1.68	6wurws	*Bacteroides ovatus*	3.77 ± 5.65
26	*Bacteroides ovatus*	0.99 ± 1.16	cbl07c	*Bacteroides ovatus*	1.66 ± 3.19
53	*Bacteroides plebeius*	0.56 ± 1.58	tmdvof	*Bacteroides plebeius*	3.07 ± 8.09
46	*Clostridium leptum*	0.56 ± 1.31	t9mxq7	*Bacteroides vulgatus*	1.20 ± 2.44
32	*Mollicutes*	0.35 ± 1.34	2uaio6	*Bacteroides vulgatus*	4.96 ± 6.85
79	*Muribaculaceae*	0.15±0.39	ff0431	*Bacteroides xylanisolvens*	3.14 ± 6.70
5	*Ruminococcus bromii*	0.87 ± 1.37	4c03f6	*Bacteroides xylanisolvens*	4.82 ± 9.40
41	*Eubacterium oxidoreducens*	0.43 ± 0.48	p3xfey	*Paraprevotella clara*	1.03 ± 3.71
7	*Bacteroides uniformis*	1.68 ± 2.66	2st345	*Blautia faecis*	1.90 ± 2.58
21	*Faecalibacillus* spp.	0.82 ± 0.69	6eanz7	*Blautia massiliensis*	4.20 ± 10.41
			hcf4f5	*Blautia obeum*	2.01 ± 3.63
			e9ptub	*Blautia wexlerae*	1.89 ± 2.30
			fepklg	*Dorea longicatena*	4.49 ± 10.19
			rfal2b	*Fusicatenibacter saccharivorans*	1.90 ± 1.50
			jbtn3b	*Lachnospiraceae* sp.	1.40 ± 1.74
			4ma8bd	*Eubacterium rectale*	1.71 ± 2.55
			3ur8h3	*Escherichia*/*Shigella* spp.	15.59 ± 28.82

[1]Bacterial operational taxonomic units (OTUs) significantly affected by arabinoxylan (AX) (q<0.15, change relative to baseline).

[2]Most abundant bacterial amplicon sequence variants (ASVs) after 6-hr anaerobic incubation with AX (average relative abundance >1.0%).

Relative abundance was assessed at six weeks of AX consumption. Data presented as mean ± SD.

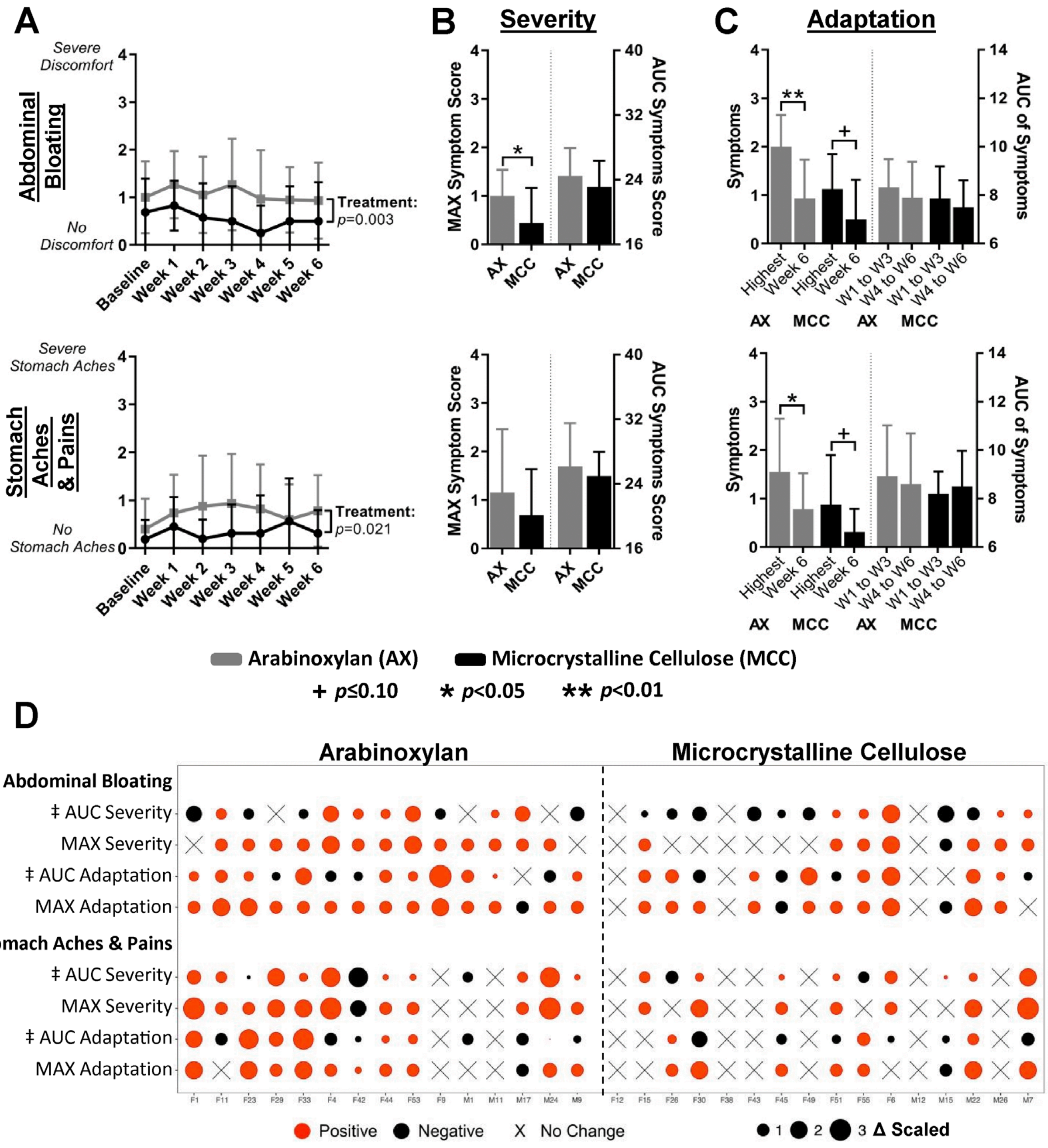

Figure S1. Characterizing the severity and adaptation of bloating and stomach ache symptoms during AX and MCC consumption. (A) Bloating and stomach aches during AX and MCC consumption (refer to **Figure 2** for overall symptoms, flatulence, and composite symptoms data). **(B)** MAX and AUC symptom score for AX and MCC. **(C)** Highest symptom during weeks

1 to 5 and week 6 symptoms for AX and MCC, as well as the AUC of symptoms from weeks 1 to 3 and weeks 4 to 6 (refer to **Supplementary Figure S3** for explanation of scores). Data in **(A)** were analysed using GEE models with Bonferroni corrections, in **(B)** using Mann-Whitney tests, and in **(C)** using Wilcoxon tests with Bonferroni corrections. Data reported as mean ± SD. Statistical significance considered at $p<0.05$. **(D)** Individualized bloating and stomach ache severity and adaptation scores for AX and MCC. Red circles represent an increase; black circles represent a decrease, and 'X' represents no change in the feature during the intervention. The circle size is proportional to the scaled magnitude change relative to baseline or week 6. ‡ Feature scaled by ($AUC_{severity}$–24/SD) or ($AUC_{adaptation}$–1/SD). AUC, area under the curve.

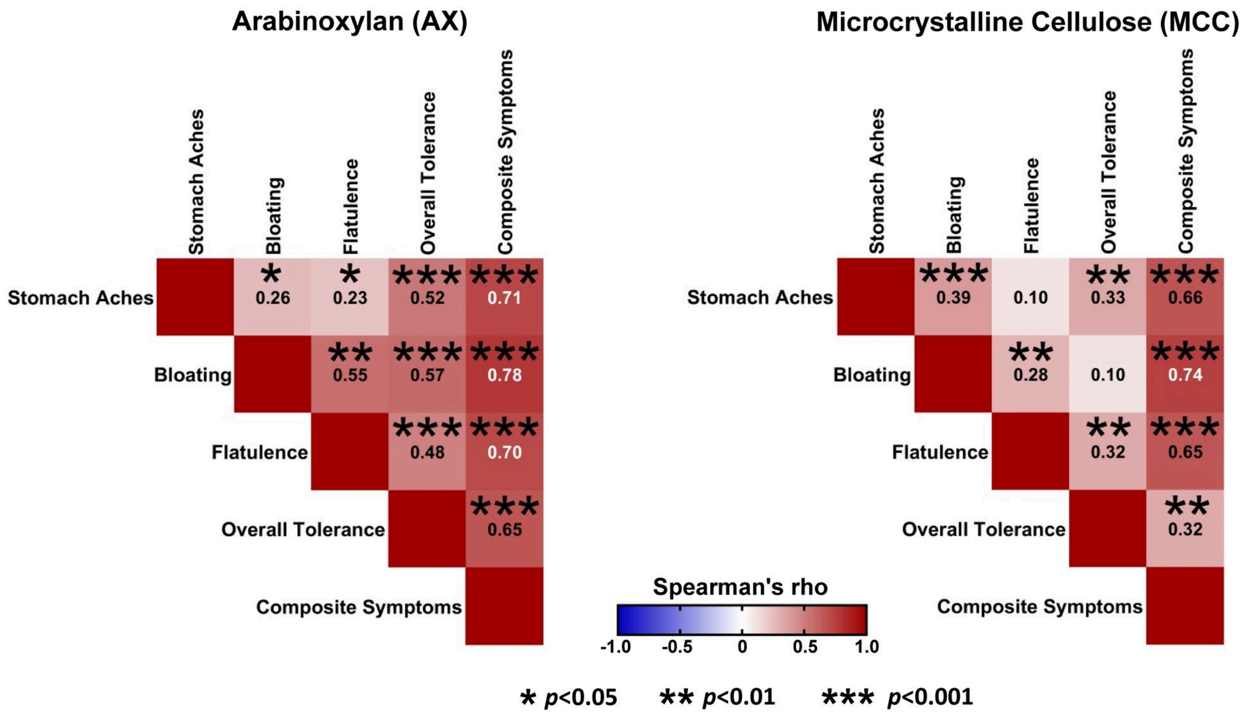

Figure S2. Multicollinearity detected between treatment-induced changes in assessed gastrointestinal symptoms. Heatmaps show Spearman's correlations between changes in individual and composite symptoms (Δ weeks 1 to 6) during AX and MCC consumption.

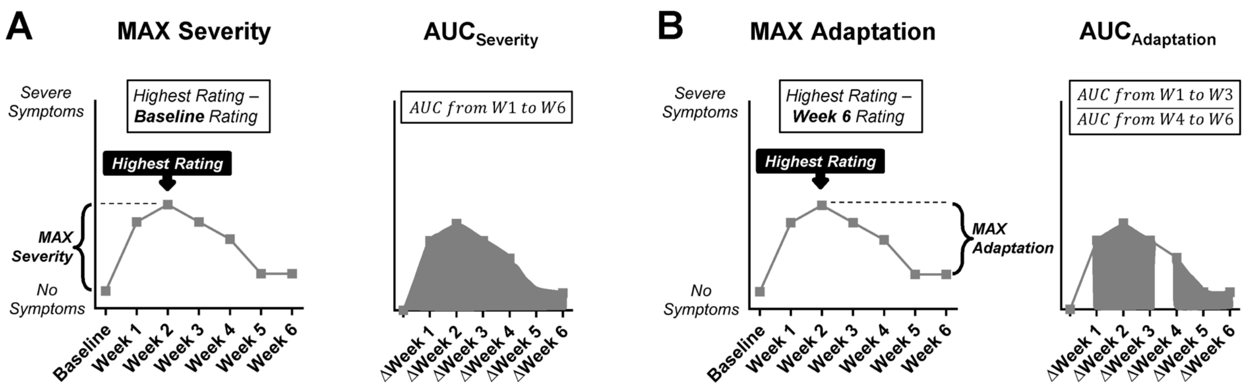

Figure S3. Graphical representation of how severity and adaptation scores were determined. (A) MAX severity scores reflect the highest score between weeks 1 to 5 minus baseline, where higher scores equal more intense symptoms. $AUC_{severity}$ scores reflect the AUC from weeks 1 to 6, where higher scores equal more severe symptoms during the intervention relative to baseline. **(B)** MAX adaptation scores reflect the highest score between weeks 1 to 5 minus week 6, where higher scores equal less intense symptoms during the final week of treatment. $AUC_{adaptation}$ scores reflect the AUC from weeks 1 to 3 divided by the AUC from weeks 4 to 6, where higher scores equal less severe symptoms during the final three weeks of treatment. AUC, area under the curve.

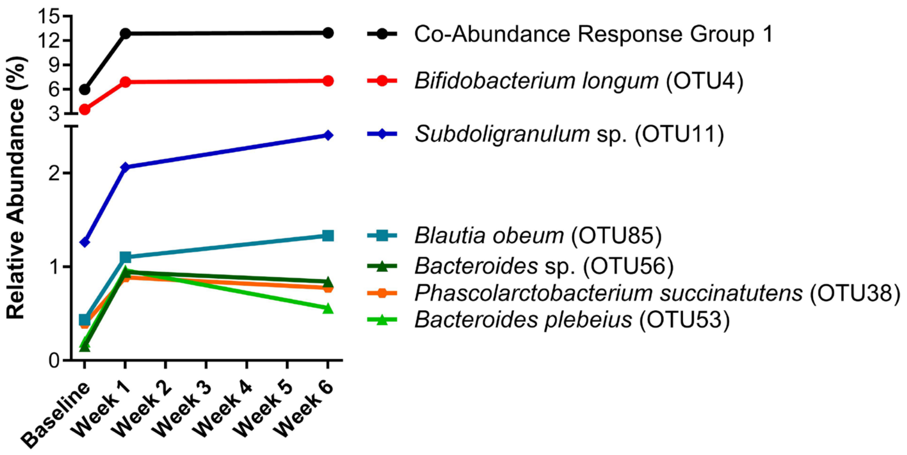

Figure S4. Dominant CARG enriched during AX consumption. Mean relative abundance of CARG1 and the encompassed group of inter-correlated OTUs that significantly increased during AX consumption (q<0.15, change relative to baseline). AX, arabinoxylan; CARG, co-abundance response group; OTU, operational taxonomic unit.

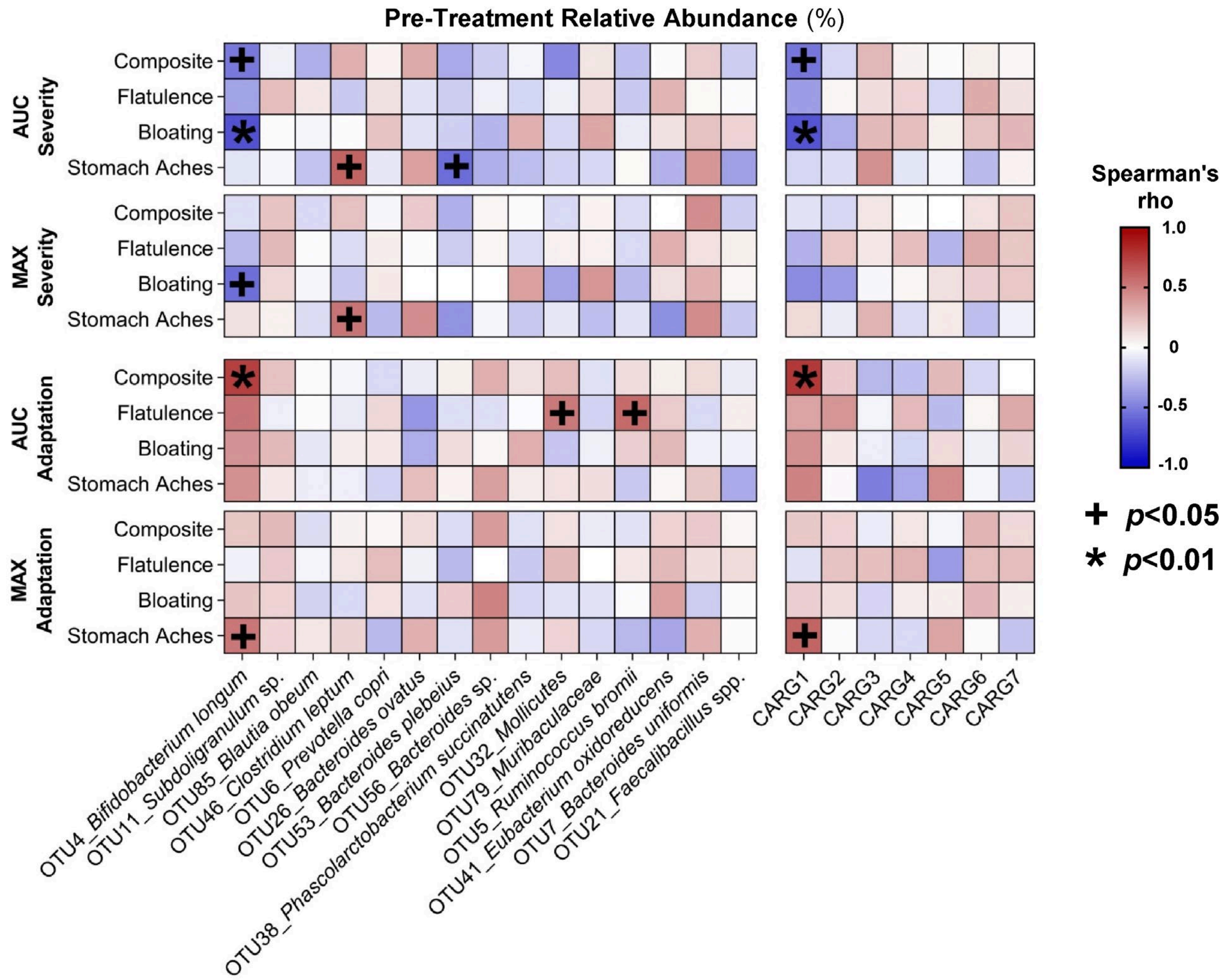

Figure S5. Associations between severity and adaptation scores and pre-treatment abundances of AX-responsive bacterial taxa in fecal samples. Heatmaps show Spearman's correlations between MAX and AUC severity and adaption scores and the pre-treatment relative abundances of all CARGs and those OTUs significantly affected by AX. Statistical significance was considered at $p<0.01$. AUC, area under the curve; AX, arabinoxylan; CARG, co-abundance response group; OTU, operational taxonomic unit.

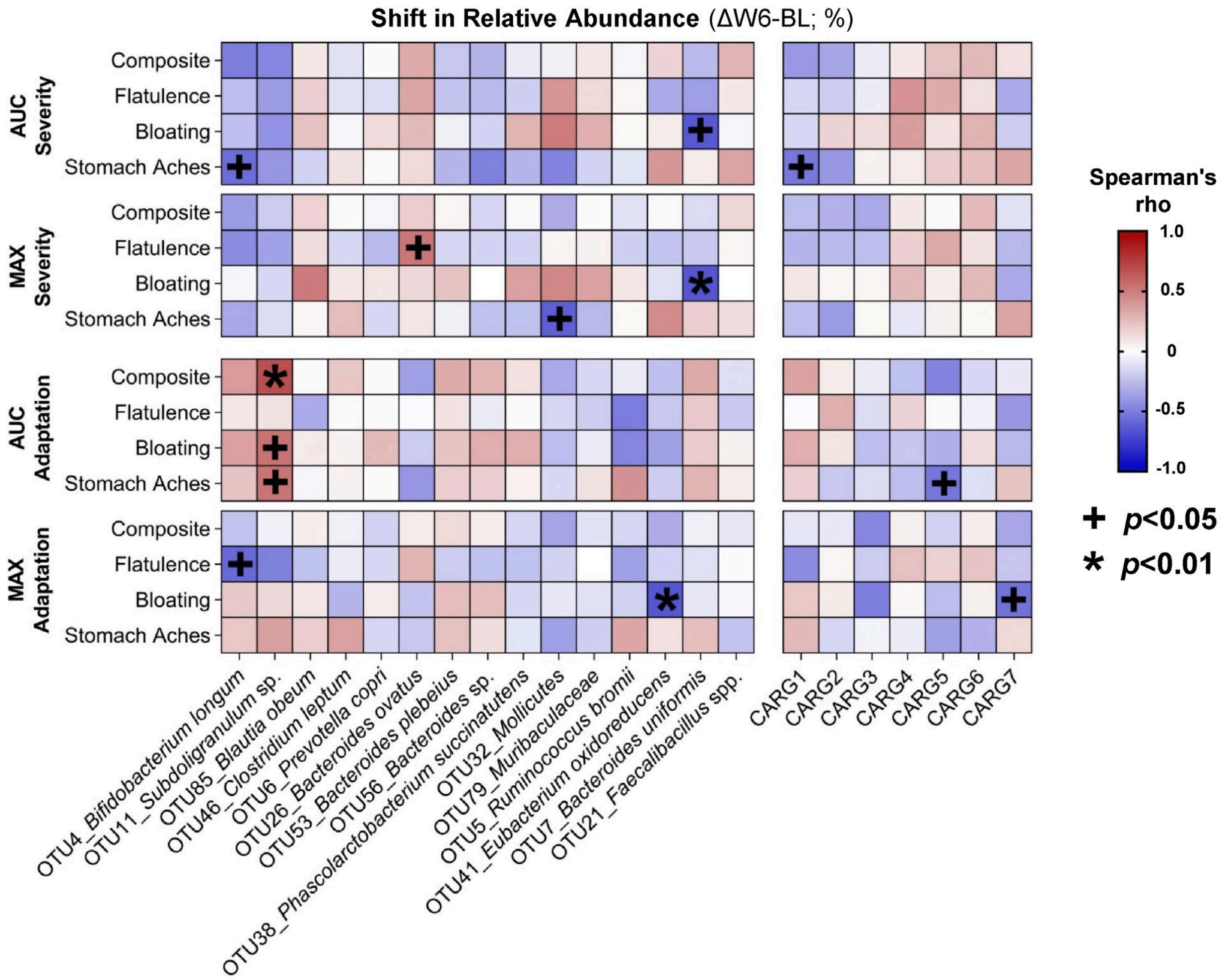

Figure S6. Severity and adaptation scores correlated with the shifts in AX-responsive bacterial taxa in fecal samples. Heatmaps show Spearman's correlations between MAX and AUC severity and adaption scores and shifts (week 6 – baseline) in the relative abundance of all CARGs and those OTUs significantly affected by AX. Statistical significance was considered at $p<0.01$. AUC, area under the curve; AX, arabinoxylan; CARG, co-abundance response group; OTU, operational taxonomic unit.

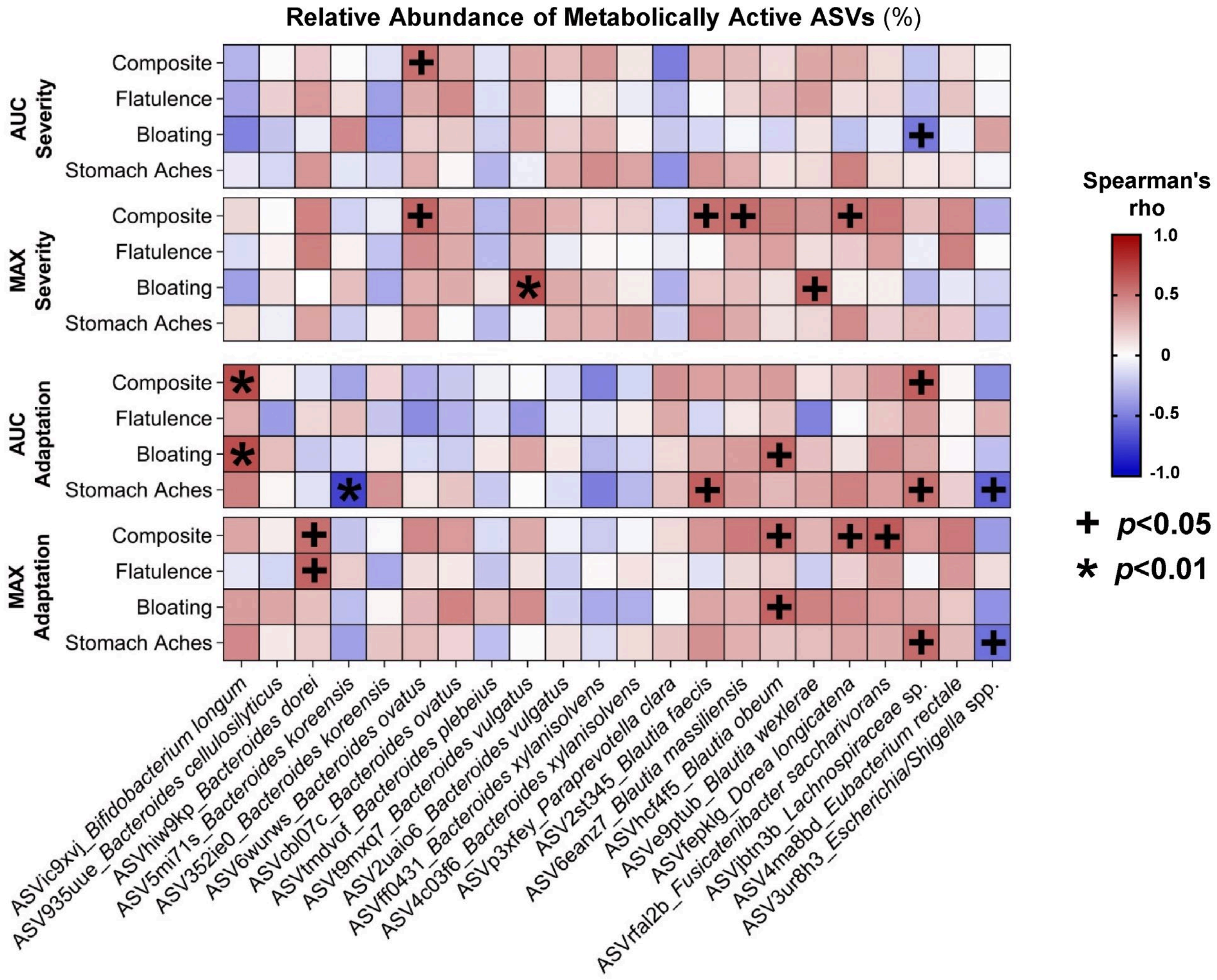

Figure S7. Associations between severity and adaptation scores and the bacterial taxa that utilise AX *ex vivo*. Heatmaps show Spearman's correlations between MAX and AUC severity and adaption scores and the relative abundance of those ASVs most metabolically active during incubation with AX (average relative abundance >1.0%). Statistical significance was considered at $p<0.01$. ASV, amplicon sequence variant; AUC, area under the curve; AX, arabinoxylan.

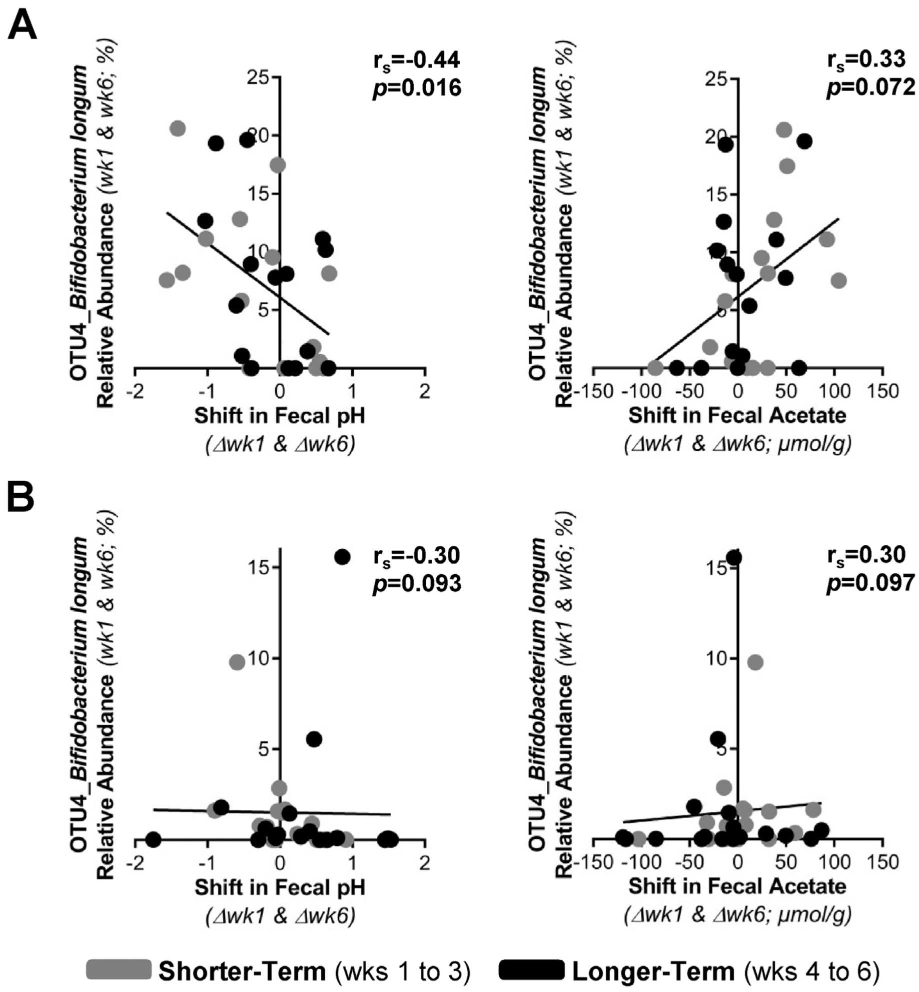

Figure S8. Associations between *Bifidobacterium longum* abundance and fecal pH and acetate shifts during AX and MCC consumption. Spearman's correlations between the relative abundance of *Bifidobacterium longum* (OTU4) (weeks 1 and 6) and fecal pH and acetate shifts (Δ weeks 1 and 6) during **(A)** AX and **(B)** MCC consumption. Gray and black dots specifying shorter- (weeks 1 to 3) and longer- (weeks 4 to 6) term time points, respectively. The best-fitting line is the linear regression line. Statistical significance was considered at $p<0.05$. AX, arabinoxylan; MCC, microcrystalline cellulose; OTU, operational taxonomic unit.

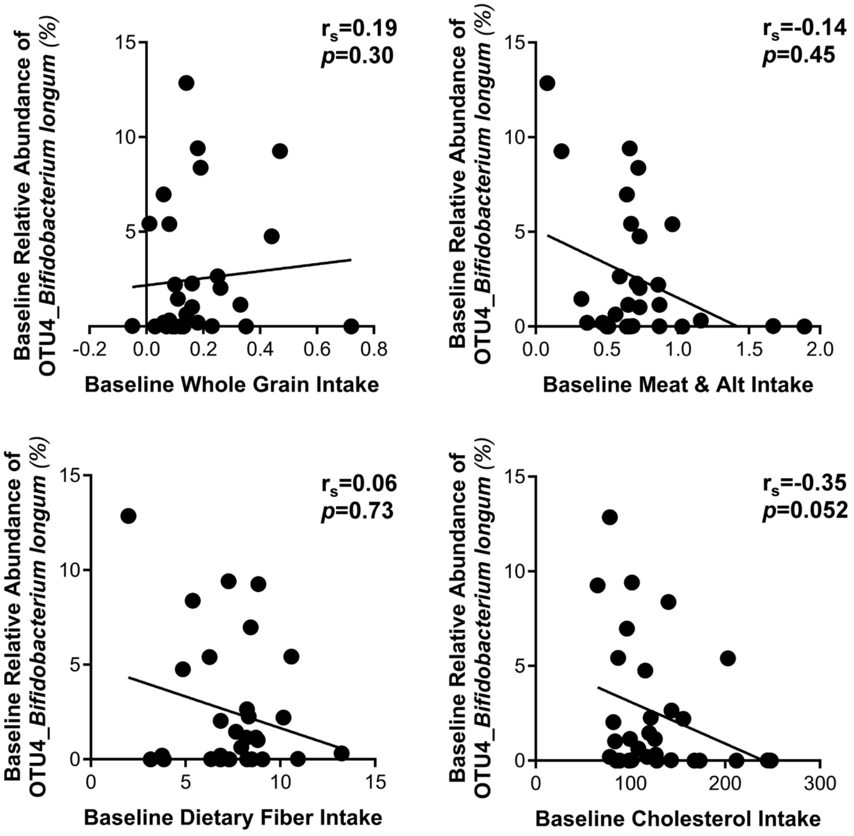

Figure S9. Associations between baseline *Bifidobacterium longum* abundance and baseline diet history. Spearman's correlations between the baseline relative abundance of *Bifidobacterium longum* (OTU4) and baseline calorie-adjusted intakes of whole grains, meat/meat alternatives, dietary fiber, and cholesterol (both AX and MCC groups included). The best-fitting line is the linear regression line. Statistical significance was considered at $p<0.05$. AX, arabinoxylan; MCC, microcrystalline cellulose; OTU, operational taxonomic unit.
